# Exploring the association between nutrition knowledge and generalized anxiety disorder among young adults in the Kingdom of Saudi Arabia: implications for Saudi Vision 2030

**DOI:** 10.3389/fnut.2025.1532394

**Published:** 2025-03-21

**Authors:** Mai Adil Ghabashi

**Affiliations:** Clinical Nutrition Department, Faculty of Applied Medical Sciences, Umm Al-Qura University, Makkah, Saudi Arabia

**Keywords:** nutrition knowledge, mental health, generalized anxiety disorder, young adults, Saudi Arabia

## Abstract

**Objectives:**

Anxiety is the most prevalent mental health issue in the Kingdom of Saudi Arabia (KSA), particularly among young adults, who face significant challenges in managing it, such as social stigma surrounding mental health, which may also discourage them from seeking help. Within the context of Saudi Vision 2030, addressing challenges and promoting the overall health and psychological wellbeing of the population is a vital public health objective. Consequently, it is suggested that exploring the potential role of nutrition knowledge as a strategy to promote overall health and wellbeing warrants further investigation. To address this gap, the present study aimed to examine the association between nutrition knowledge and distinct factors, including Generalized Anxiety Disorder (GAD), among young adults in the KSA.

**Methods:**

This cross-sectional study included 444 participants. Validated questionnaires assessing nutrition knowledge and specific aspects of mental health were administered in Arabic. Linear and logistic regression analyses were then employed to examine the associations between nutrition knowledge and various aspects of mental health.

**Results:**

It was found that 51.3% of the participants did not have a satisfactory level of nutrition knowledge, while 35.1% were affected by GAD. Notably, a high level of nutrition knowledge emerged as a protective factor against the risk of developing GAD among young Saudi adults. Specifically, the probability of developing GAD was 40% lower among youth with satisfactory nutrition knowledge, compared to those with unsatisfactory knowledge (OR = 0.6, 95% CI; 0.3 to 0.9; *p* = 0.02). Furthermore, the completion of nutrition-related courses emerged as a predictive factor for having a satisfactory level of nutrition knowledge. Individuals who had completed nutrition courses were ~4.6 times (95% CI; 2.9–7.4; *p* < 0.001) more likely to demonstrate a satisfactory level of nutrition knowledge, in comparison to those who had not taken such courses.

**Conclusion:**

To the best of my knowledge, this study is the first to investigate the association between nutrition knowledge and GAD in young Saudi adults. The findings suggest that the implementation of targeted nutrition education interventions may serve as a promising strategy to enhance overall health and wellbeing among the young population living in the KSA. Utilizing technology and social media may facilitate the delivery of these interventions, making them more accessible and engaging for young Saudi individuals.

## 1 Introduction

Enhancing overall health and psychological wellbeing represents a major public health priority within the framework of Saudi Vision 2030 in the Kingdom of Saudi Arabia (KSA) (1). This comprehensive reform agenda, initiated by the Saudi government, seeks to improve community health, augment social wellbeing and foster economic transformation (2, 3). However, the increased prevalence of mental health problems in the KSA constitutes a substantial barrier to the achievement of optimal mental health and overall wellbeing (4). According to the latest Saudi National Mental Health Survey, the prevalence of mental disorders in the Kingdom of Saudi Arabia stands at 20.2%. Among these disorders, anxiety is the most prevalent, affecting 12.3% of the population, especially young adults who are the most affected group (5). Hence, it is important to consider strategies that help in reducing anxiety levels among the Saudi population to achieve the objectives outlined in Saudi Vision 2030.

Despite the availability of various techniques for managing anxiety among young adults, such as cognitive-behavioral therapy (CBT) (6) and mindfulness-based stress reduction (MBSR) (7), several challenges hinder their effectiveness. One significant barrier is the stigma associated with mental health issues, which often discourages young adults from seeking help or engaging with these interventions. Additionally, concerns about confidentiality and lack of awareness regarding the benefits and availability of mental health resources contribute to their underutilization (8). Addressing these barriers is crucial for improving the effectiveness and accessibility of anxiety management techniques for young adults.

In contrast to such therapeutic approaches, improving nutrition knowledge (NK) may emerge as a key factor for achieving objectives related to the health improvement aspect of Saudi Vision 2030, which prioritizes both physical and mental wellbeing (9). It is often more accessible and less stigmatized, making it a viable complementary strategy for promoting overall wellbeing. Adequate nutrition knowledge may empower individuals to make informed dietary choices that support overall health and wellbeing (10, 11). Awareness of the significance of essential nutrient intake and balanced diet consumption enables individuals to meet their nutritional requirements effectively (12, 13). This can enhance the functioning of bodily systems, strengthen the immune system, and promote optimal organ function (14, 15). Interestingly, not only does adequate nutrition knowledge play a vital role in promoting overall physical health, but it also has the potential to positively impact psychological wellbeing within the population (16).

Emerging evidence indicates a potential relationship between good nutrition knowledge and a reduction in anxiety symptoms (17). This might be attributed to the consequent consumption of healthy foods associated with satisfactory nutrition knowledge. For instance, a previous short-term intervention study investigated the influence of daily healthy snacks (such as fruits) vs. unhealthy snacks (such as chocolate and chips) on mental health. The study involved 100 college student volunteers and lasted for 10 days. During this period, the group that received the healthy snacks experienced a reduction in anxiety levels compared to the group that consumed the unhealthy snacks (18). Similar findings were reported by another 12-week intervention focused on increasing adherence to a Mediterranean diet among 60 volunteers. This study found a significant reduction in anxiety scores in the intervention group compared to the control group (19). Although the mechanism of nutrients affecting mental health is not yet fully understood, it is suggested that certain nutrients may play a vital role in supporting brain function and influencing neurotransmitter production, which are essential factors in maintaining mental wellbeing (20). However, further research is required to enhance our understanding of these associations.

Uunderstanding the association between nutrition knowledge and GAD among young adults is of significant importance from a public health perspective. By exploring this relationship, policymakers and public health practitioners can develop targeted interventions and educational programs to enhance nutrition knowledge, promote healthy dietary choices, and potentially reduce the burden of GAD on this population. To the best of my knowledge, there is a lack of studies examining the association between nutrition knowledge and GAD in the KSA. Therefore, this research aims to bridge this gap in the literature by exploring such associations.

## 2 Materials and methods

### 2.1 Study design

**Figure 1:**
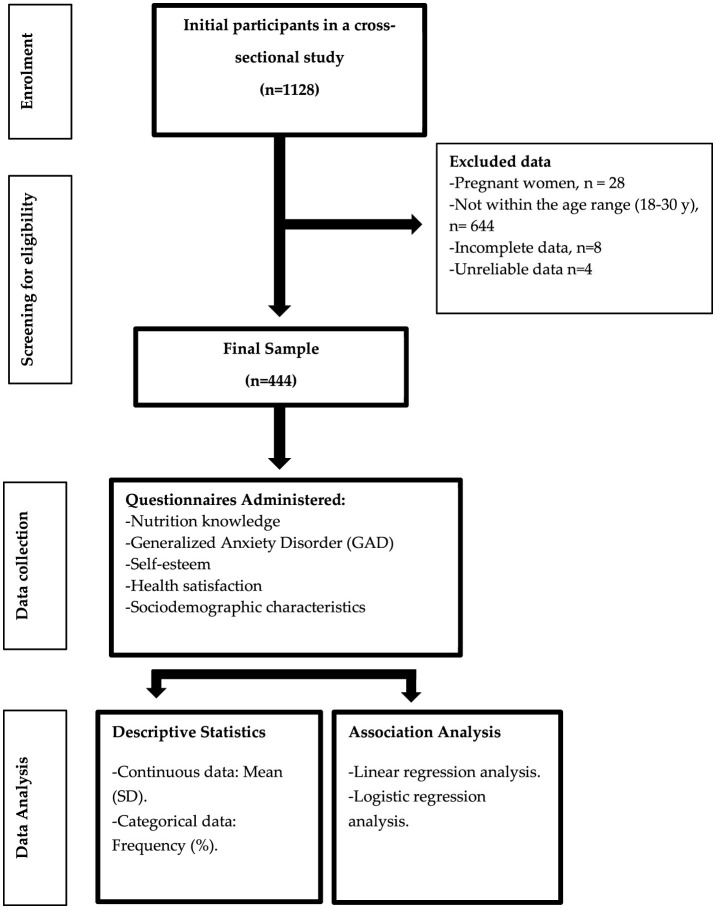
Overview of participant enrolment and study methodology.

A cross-sectional study was conducted between June and September 2024 using an anonymous online survey administered through Google Forms. The survey was distributed via the snowball sampling technique, utilizing social media platforms as a means of dissemination. Through the digital interface of this platform, and prior to participation, it was explicitly stated that participation is voluntary, that the survey would be anonymous, and no personally identifiable information would be collected. Participants were assured that all data handling and analysis would be treated with strict confidentiality. Only those participants who provided informed consent proceeded to the main sections of the survey. A flow diagram of participant selection and research methodology is presented in Figure 1.

### 2.2 Sample size

Sample size calculation was performed using RaoSoft, a digital sample size calculator. The calculation determined that a minimum of 377 participants in total were needed to conduct the study with a 95% confidence interval and a 5% margin of error. The study reached a wider range of the population, where 1,128 participants completed the questionnaire. Of those, 444 met the inclusion criteria of the study and their responses were analyzed. The inclusion criteria for this study consisted of participants aged 18–30 years old residing in the KSA who read and write in Arabic. The exclusion criteria encompassed individuals who did not fall within the defined young adulthood age range, pregnant women, and those who provided incomplete or invalid responses.

### 2.3 Study tools and data collection

Data collection included the basic sociodemographic characteristics, such as age, sex, marital status, etc. Additionally, the participants' self-reported weight and height were used to calculate their body mass index (BMI). Specifically, BMI was calculated by dividing each individual's weight in kilograms by their height in squared meters. Based on these BMI values, the participants were then categorized into standard weight status groups: underweight (<18.5 kg/m^2^), normal weight (18.5–24.9 kg/m^2^), overweight (25.0–29.9 kg/m^2^), and obesity (30.0 kg/m^2^ or greater). Moreover, the questionnaire collected data about nutrition knowledge, and variables related to psychological wellbeing. More details are provided below.

#### 2.3.1 Nutrition knowledge

Nutrition knowledge was assessed using a validated dietary questionnaire on nutrition knowledge, which was distributed in Arabic language (21). The questionnaire consisted of 10 questions, with a maximum score of 10 points. Each correct answer was assigned a score of 1, while incorrect answers or uncertainty were given a score of 0. The final score was obtained by summing the individual scores. To classify the level of nutrition knowledge, modified Bloom's cut-off points were utilized. Specifically, a score of 80%−100% of correct responses (8–10 points) indicated a good level of nutrition knowledge. A score of 50%−79% (5–7.9 points) represented a moderate level of nutrition knowledge, while a score below 50% of correct responses (<5 points) denoted a low level of nutrition knowledge. To facilitate the statistical analysis, those categories were dichotomized into two groups: satisfactory nutrition knowledge (>80% of correct answers) and unsatisfactory nutrition knowledge (<80% of correct answers) (22, 23).

#### 2.3.2 General Anxiety Disorder (GAD)

General Anxiety Disorder (GAD) was assessed using a 7-item validated questionnaire (24). It is a widely used tool for screening and measuring the severity of GAD. The Arabic version of this questionnaire has been adopted by the Saudi Arabian Ministry of Health as a self-administered tool for the initial screening of anxiety levels (25). The GAD-7 questions assess various signs of GAD. Each item was scored on a 4-point Likert scale ranging from 0 (not at all) to 3 (nearly every day). The total score can range from 0 to 21, with higher scores indicating higher levels of anxiety. The severity levels of anxiety were classified as follows: a score of 0–4 indicated minimal anxiety, 5–9 denoted mild anxiety, 10–14 represented moderate anxiety, and a score of 15–21 indicated severe anxiety. To facilitate analysis, a cutoff point of ≥10 was established to signify the presence of anxiety (26).

#### 2.3.3 Self-esteem

Self-esteem was assessed using the validated Arabic version of the single-item self-esteem scale (27), which asked participants to rate the statement “I have high self-esteem.” The scale utilized a 5-point Likert scale ranging from “not at all true of me” to “very true of me.” The coding was as follows (1 = not at all true of me, 2 = rather not true of me, 3 = some part true of me, 4 = rather true of me, 5 = very true of me). Participants selected the response option that best reflected their agreement or disagreement with the statement. A higher score indicated higher self-esteem, and a lower score indicated reduced self-esteem. Answers were dichotomized into two categories: “Low self-esteem” (including responses 1–3) and “High self-esteem” (including responses 4 and 5) (28).

#### 2.3.4 Health satisfaction

Health satisfaction was assessed using the question, “How satisfied are you with your health?”. This question was derived from the validated Arabic version of the World Health Organization Quality of Life Questionnaire. Participants were provided with a Likert scale consisting of five response categories: (1) very dissatisfied, (2) dissatisfied, (3) neither dissatisfied nor satisfied, (4) satisfied, and (5) very satisfied (29). Responses were dichotomized into two categories: “Low health-satisfaction” (including responses 1 to 3) and “High health-satisfaction” (including responses 4 and 5) (28).

### 2.4 Statistical analysis

The data analysis was performed using IBM SPSS (version 29, Chicago, IL, USA). Continuous data were presented as mean (SD), while categorical data were presented as frequency (%). Multivariate linear regression analyses were utilized to examine the relationship between nutrition knowledge scores and a range of independent variables, providing a quantitative assessment of the strength and direction of this association. Standardized beta coefficients were used to estimate the magnitude of the effect of each independent variable on the nutrition knowledge scores, taking into account the influence of other variables in the model. The independent variables in the model accounted for 20% of the variance in the nutrition knowledge score (adjusted *r*^2^ = 0.20). To further investigate the relationship between the variables, a subsequent analysis was performed with the score of GAD as the dependent variable. The analysis yielded adjusted *r*^2^ value of 0.23, indicating that ~23% of the variance in GAD scores could be explained by the independent variables in the model. While these values suggest a modest level of explanatory power, they highlight the complexity of the factors influencing nutrition knowledge.

Additionally, multivariate logistic regression analysis was employed to evaluate the association between achieving a satisfactory level of nutrition knowledge and other variables. The odds ratio (OR) was used to estimate the probability of achieving a satisfactory level of nutrition knowledge in relation to different characteristics. The Hosmer–Lemeshow goodness-of-fit test was conducted, and the obtained *p*-value was 0.3, indicating a good fit of the logistic regression model to the data. To enhance our understanding, a follow-up analysis was performed with the presence of GAD as the dependent variable. This model also demonstrated a good fit, as indicated by a Hosmer–Lemeshow test *p*-value of 0.6. In both the linear regression and logistic regression analyses, a 95% confidence interval (CI) was calculated for the estimated coefficients (beta coefficients in linear regression and odds ratios in logistic regression). The 95% CI indicates a range of values within which the true population parameter is expected to lie with 95% confidence. Level of significance was set at *p* < 0.05.

## 3 Results

The study comprised of 444 young participants residing in the KSA, with a mean age of 22.0 ± 3.4 years. The sample exhibited a predominant representation of female participants, constituting 90.8% (403/444) of the total sample size. The majority of participants 87.8% (390/444) were Saudi Arabian (see Table 1 for a full breakdown of the study participants' sociodemographic characteristics). The prevalence of overweight was 19.4% (86/444), while the prevalence of obesity was 16.7% (74/444) in the study population. Remarkably, over one-third of the participants were diagnosed with GAD, with 24.3% (108/444) exhibiting a moderate level of GAD and 10.8% (48/444) demonstrating severe symptoms (Table 2).

**Table 1:** Sociodemographic characteristics of study participants.

**Variable**	**Participants (*****n*** = **444)**	
Age, years	22.0	3.4
Early young adults (18–24 years)	362.0	81.5
Late young adults (25–30 years)	82.0	18.5
**Sex**, ***n*****%**		
Male	41.0	9.2
Female	403.0	90.8
**Nationality, n%**		
Saudi	390.0	87.8
Non-Saudi	54.0	12.2
**Social status**, ***n*****%**		
Single	379.0	85.4
Married	65.0	14.6
**Educational status**, ***n*****%**		
Student	349.0	78.6
Graduated	95.0	21.4
**Completion of nutrition courses**, ***n*****%**		
Yes	138.0	31.1
No	306.0	68.9
**Interest in attending nutrition educational courses**, ***n*****%**		
Interest in face-to-face courses	126.0	14.5
Interest in digital courses	370.0	42.7
No interest	371.0	42.8
BMI, kg/m^2^	23.8	5.8
**Body weight**, ***n*****%**		
Underweight	73.0	16.4
Normal	211.0	47.5
Overweight	86.0	19.4
Obesity	74.0	16.7
**Health status**		
Healthy	359.0	80.9
Diagnosed with a disease	85.0	19.1
Total nutrition knowledge score	7.2	1.7

All data are mean and standard deviation unless indicated.

**Table 2:** Psychological characteristics of study participants.

**Variable**	**Overall (*****n*** = **444)**	
GAD score	8.3	4.3
**GAD severity**, ***n*** **(%)**		
Minimal	98.0	22.1
Mild	190.0	42.8
Moderate	108.0	24.3
Severe	48.0	10.8
Self-esteem score	3.8	0.9
**Level of self-esteem**, ***n*** **(%)**		
High	285.0	64.2
Low	159.0	35.8
Health satisfaction score	3.34	0.5
**Level of health satisfaction**, ***n*** **(%)**		
High	219.0	49.3
Low	225.0	50.7

All data are mean and standard deviation unless indicated.

**Figure 2:**
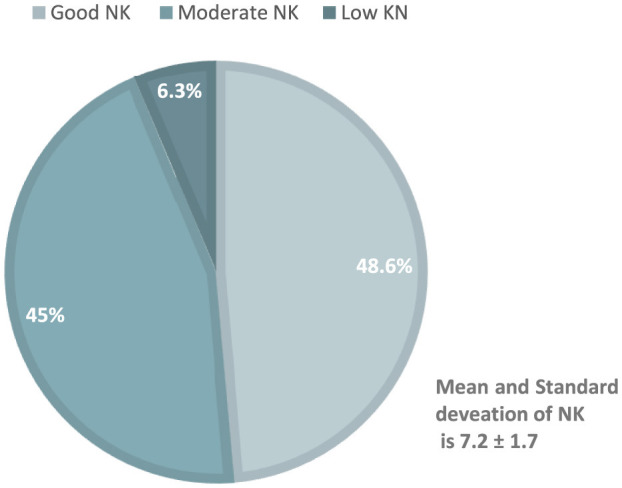
Nutrition knowledge of the study participants.

The mean nutrition knowledge score was 7.2 ± 1.7. Upon categorization, the distribution of nutrition knowledge levels was as follows: 48.6% (216/444) demonstrated good nutrition knowledge, 45% (200/444) exhibited moderate nutrition knowledge, and 6.3% (28/444) had low nutrition knowledge (Figure 2). For the purpose of analysis, these categories were subsequently reconfigured into a binary classification: participants with a satisfactory level of nutrition knowledge accounted for 49% (201/444), while those with an unsatisfactory level of nutrition knowledge constituted 51% (228/444) of the sample. Notably, more than half of the participants demonstrated an interest in enrolling on nutrition education courses. Specifically, 42.7% (370/444) of the participants expressed a favorable inclination toward utilizing digital means to attend such courses, while 14.5% (126/444) preferred traditional face-to-face educational sessions (Table 1). The associations between nutrition knowledge (as the dependent variable) and various independent variables were examined using linear and logistic regression analyses, as detailed in Tables 3, 4 respectively. To provide a better understanding and clarity of such associations, a follow-up analysis was conducted, with GAD serving as the dependent variable, as shown in Tables 5, 6.

**Table 3:** Associations between nutrition knowledge score and study participants' characteristics: linear regression analysis.

**Variables**	**β**	**(95% CI)**	***p***
Age, years	0.1	(0.1, −0.0)	0.2
Nationality, Saudi	0.1	(0.1, 0.1)	**0.008**
Gender, female	−0.0	(−0.0, −0.6)	0.6
Social status, married	0.1	(0.1, −0.1)	0.1
Education, graduated	0.0	(0.0, −0.3)	0.5
Completion of Nutrition courses	0.4	(0.4, 1.1)	<**0.001**
Health status, diagnosed with a disease	0.1	(0.1, −0.1)	0.12
BMI, kg/m^2^	0.1	(0.1, −0.0)	0.2
Generalized Anxiety Disorder score	−0.2	(−0.1, −0.0)	**0.002**
Self-esteem score	0.1	(−0.0, 0.2)	0.1
Health satisfaction score	−0.1	(−0.3, −0.0)	**0.04**

β, beta coefficient; CI, confidence interval.

Nutrition Knowledge score is the dependent variable. Linear regression analysis metrics: r = 0.45, adjusted r^2 =^ 0.20, F = 10.40, *p* < 0.001. Entries presented in bold signify a statistically significant level, with a *p*-value < 0.05.

**Table 4:** Associations between satisfactory level of nutrition knowledge and study participants' characteristics: logistic regression analysis.

**Variables**	**OR**	**(95% CI)**	***p***
Age, years	1.0	(0.9, 0.1)	0.6
Nationality, Saudi	1.5	(0.8, 3.0)	0.1
Gender, female	1.0	(0.5, 2.2)	0.8
Social Status, married	1.1	(0.5, 2.1)	0.7
Education, graduated	1.2	(0.6, 2.4)	0.4
Completion of nutrition courses	4.6	(2.9, 7.4)	<**0.001**
Health status, diagnosed with a disease	2.0	(1.1, 3.5)	**0.01**
Body weight status, overweight/obese	0.0	(0.0, 0.0)	0.9
Generalized Anxiety Disorder, moderate to severe	0.5	(0.3, 0.8)	**0.01**
High self-esteem	1.1	(0.7, 1.7)	0.6
High health satisfaction	0.9	(0.5, 1.3)	0.6

OR, odds ratio; CI, confidence interval.

Satisfactory Level of Nutrition Knowledge is the dependent variable. Logistic regression analysis metrics: Hosmer–Lemeshow Test *p* = 0.3, Nagelkerke R^2^ = 0.20, −2 log likelihood = 448.32. Entries presented in bold signify a statistically significant level, with a *p*-value < 0.05.

**Table 5:** Associations between generalized anxiety disorder score and study participants' characteristics: linear regression analysis.

**Variables**	**β**	**(95% CI)**	***p***
Age, years	−0.2	(−0.4, −0.1)	<**0.001**
Nationality, Saudi	0.0	(−0.9, 1.4)	0.6
Gender, female	0.1	(−0.1, 2.7)	0.07
Social status, married	−0.1	(−2.4, 0.0)	0.05
Education, graduated	−0.0	(−1.4, 1.0)	0.7
Completion of Nutrition courses	0.0	(−1.2, 3.2)	0.6
Health status, diagnosed with a disease	0.2	(1.2, 3.2)	<**0.001**
BMI, kg/m^2^	−0.2	(−0.0, 0.1)	0.1
Nutrition Knowledge score	−0.1	(−1.2, −0.4)	**0.002**
Self-esteem score	−0.2	(−1.2, −0.4)	<**0.001**
Health satisfaction score	−0.2	(−1.2, −0.4)	<**0.001**

β, beta coefficient; CI, confidence interval.

GAD score is the dependent variable. Linear regression analysis metrics: r = 0.48, adjusted r^2 =^ 0.23, F = 11.94, *p* < 0.001. Entries presented in bold signify a statistically significant level, with a *p*-value < 0.05.

**Table 6:** Associations between the existence of generalized anxiety disorder and study participants' characteristics: logistic regression analysis.

**Variables**	**OR**	**(95% CI)**	***p***
Age, years	0.9	(0.7, 0.9)	**0.005**
Nationality, Saudi	1.1	(0.5, 2.1)	0.7
Gender, female	1.8	(0.7, 4.1)	0.1
Social Status, married	0.7	(0.3, 1.4)	0.3
Education, graduated	1.2	(0.5, 2.3)	0.6
Completion of Nutrition courses	1.1	(0.6, 1.8)	0.7
Health status, diagnosed with a disease	3.0	(1.7, 5.0)	<**0.001**
Body weight status, overweight/obese	1.0	(0.6, 1.5)	0.9
Satisfactory level of Nutrition Knowledge	0.6	(0.3, 0.9)	**0.02**
High self-esteem	0.4	(0.2, 0.6)	<**0.001**
High health satisfaction	0.5	(0.3, 0.7)	**0.002**

OR, odds ratio; CI, confidence interval.

The existence of GAD is the dependent variable.

Logistic regression analysis metrics: Hosmer–Lemeshow Test *p* = 0.6, Nagelkerke R^2^ = 0.23, −2 log likelihood = 495.47. Entries presented in bold signify a statistically significant level, with a *p*-value < 0.05.

The linear regression analysis found a positive significant association between Saudi nationality and nutrition knowledge score (β = 0.1, 95% CI; 0.1 to 0.1; *p* = 0.008). Additionally, the completion of nutrition courses was associated with higher nutrition knowledge scores (β = 0.4, 95% CI; 0.4 to 1.1; *p* < 0.001; Table 3). Participants who had completed nutrition courses were nearly 4.6 times (95% CI; 2.9 to 7.4; *p* < 0.001) more likely to have a satisfactory level of nutrition knowledge compared to those who had not (Table 4). As expected, the presence of a disease was significantly associated with a higher likelihood of possessing satisfactory nutrition knowledge. Participants with health issues exhibited a twofold increased likelihood of possessing satisfactory knowledge in the field of nutrition compared to their healthy counterparts (OR = 2.0, 95% CI; 1.1 to 3.5; *p* = 0.01; Table 4). Interestingly, nutrition knowledge score was found to be inversely associated with GAD scores among young adults in the KSA (β = −0.1, 95% CI; −1.2 to −0.4; *p* = 0.002), which indicates that for each unit increase in nutrition knowledge, the GAD score decreases by 0.1 units (Table 5). As shown in the subsequent logistic regression analysis, a satisfactory level of nutrition knowledge was associated with a reduced likelihood of developing GAD. Specifically, the probability of developing GAD was 40% lower among youth with satisfactory nutrition knowledge compared to those with unsatisfactory knowledge (OR = 0.6, 95% CI; 0.3 to 0.9; *p* = 0.02; Table 6).

## 4 Discussion

This study contributes to the understanding of the relationship between nutrition knowledge and GAD among young adults in Saudi Arabia. It offers insights into the occurrence of GAD and levels of sufficient nutrition knowledge, and examines the associations between these two variables. The findings indicate that the nutrition knowledge level was not satisfactory among more than half of the study participants. Such finding aligns with the findings of a recent extensive investigation conducted among 8,191 participants from five different Arab countries, including Saudi Arabia, Jordan, Egypt and Syria. This large-scale study reported that nearly two-thirds of the participants exhibited low or moderate levels of nutrition knowledge (22). This highlights the significance of addressing such an issue, in order to contribute to the broader objectives of Saudi Vision 2030. That is, enhancing nutrition knowledge is crucial for achieving better health outcomes within the community. To facilitate this improvement, it is recommended to incorporate both formal academic courses and community-based nutrition education programs. This recommendation is supported by another finding from the present study, which indicated that participants who had completed nutrition courses were twice as likely to demonstrate a satisfactory level of nutrition knowledge compared to those who had not. Such observation underscores the pivotal role of nutrition education in promoting satisfactory levels of nutrition knowledge among the Saudi population.

According to the findings of the current study, the probability of achieving a satisfactory nutrition knowledge level was doubled in people who were diagnosed with a disease compared to their healthy counterparts. This may potentially be explained by the fact that individuals with a higher baseline of nutrition knowledge are more proactive about their health and may be more likely to seek medical attention, leading to an earlier diagnosis of their disease. In this case, the higher nutrition knowledge precedes the disease diagnosis (30). Another explanation might be related to the concept of illness-induced motivation. This concept suggests that the experience of being diagnosed with a health condition prompts individuals to become more motivated to learn about and adopt healthier behaviors, including improving their nutrition knowledge, as a means of managing their condition (31). This supports the suggestion that nutrition knowledge plays a critical role in reducing the severity of disease symptoms. In brief, the improvement of nutrition knowledge is important for achieving the goals of Saudi Vision 2030, which seeks to promote health and wellness among the population.

Another notable finding of the current study was the high incidence of GAD observed in nearly one-third of the participants. This rate is consistent with the global range of anxiety prevalence reported in the literature. For instance, a recent systematic review and meta-analysis that included 192 studies worldwide found that the prevalence of anxiety ranges from ~38% to 73% in populations up to 25 years of age (32). An additional systematic review involving 16 studies reported that young adults aged 18–29 years old are the demographic group most prone to experiencing anxiety disorders (33). With regards to the KSA, the proportion of the study sample affected by anxiety is comparable to that reported by a review involving 19 studies conducted in the region that targeted high school and college students. That review reported a prevalence of anxiety ranging between 34.9% and 65% (34). This suggests that the high rate of anxiety observed in the current study aligns with the broader global and local trends of elevated anxiety among the young population. These results highlight the importance of considering factors that may help manage and reduce anxiety levels within the Saudi population.

Interestingly, the present study found that having a satisfactory level of nutrition knowledge was associated with lower odds of developing GAD. The results detected a 40% reduction in the likelihood of experiencing GAD among participants with satisfactory nutrition knowledge compared to their counterparts with unsatisfactory knowledge. This finding is consistent with the results of a recent study conducted in 2023, on the relationship between nutrition knowledge and mental health. The study involved university students from England and Turkey, and detected a significant inverse correlation between nutrition knowledge and indicators of mental health issues, including anxiety and depression (35). Further recent and consistent findings were reported by a study published in 2024, which involved Chinese college students. The findings indicated a significant inverse relationship; more specifically, students with high levels of nutrition literacy were 47% less likely to develop GAD compared to those with low levels of nutrition literacy. Additionally, the study found that individuals with adequate nutrition literacy had 2.52 times higher odds of experiencing a high quality of life compared to their counterparts with insufficient nutrition literacy (36). Such associations could be explained by the Social Cognitive Theory, which suggest that knowledge not only informs individuals about healthy choices, but also shapes their attitudes and beliefs about nutrition, ultimately influencing their dietary behaviors (10, 37). Improvements of dietary habits could therefore play a role in enhancing mental health (38, 39).

However, while the current study highlights the importance of nutrition knowledge, it is crucial to recognize that specific dietary habits and food intake patterns were not precisely measured. These unexamined factors may significantly mediate the observed relationship between nutrition knowledge and anxiety. For instance, the quality and quantity of food consumed can directly impact mental health outcomes. Specific food items or combinations may be particularly beneficial. For instance, a recent study involving a sample of Saudi population found that daily fruit intake was linked to nearly a threefold increase in the likelihood of achieving a satisfactory level of self-regulation in eating behavior, which, in turn, has been inversely associated with lower scores of GAD (40). This phenomenon may be attributed to fiber intake, which enhances satiety. The feeling of fullness can reduce the likelihood of overeating, thereby improving self-regulation. As individuals gain better control over their eating behaviors, they may experience reduced stress related to food choices and body image.

Moreover, a previous systematic review of 61 studies found that a high intake of fruits and vegetables, in particular, is associated with a reduction of psychological distress and increased levels of optimism (41). This is likely due to the abundant fiber content in fruits and vegetables. Recent research compared the effectiveness of a high-fiber diet with that of a high-protein diet regarding tryptophan metabolism. The results indicated that a high-fiber diet significantly enhances the production of beneficial tryptophan metabolites, including indole-3-acetic acid (IAA) and indole-3-propionic acid (IPA). This increase in metabolites subsequently raises serotonin levels (42), a key neurotransmitter involved in regulating mood, emotional stability, and overall psychological wellbeing (43). An additional study that involved Turkish students found that anxiety levels were significantly lower in those who consume foods rich in prebiotics and probiotics (44). This finding may be explained by the gut-brain axis, which highlights the influence of gut health on mental wellbeing (45). For instance, research has shown that beneficial bacteria, including *Lactobacillus acidophilus* and *Bifidobacterium bifidum*, can significantly increase levels of the hormone oxytocin (46), which is essential for fostering social connections, emotional bonding, and stress alleviation (47).

Another recent case-control study published in 2024, which focused on Iranian patients, found that adherence to healthy dietary patterns was associated with a 74% reduction in the risk of developing anxiety disorder compared to those who adopted unhealthy dietary habits (48). It is suggested that compounds, such as B vitamins, antioxidants and omega-3 fatty acids may influence neurotransmitter pathways, neuroplasticity, inflammation modulation, and oxidative stress—all of which are factors that are associated with psychological wellbeing. For example, B vitamins contribute to the production of mood-regulating neurotransmitters, while antioxidants may protect the brain from oxidative damage that can negatively affect cognition and mental health. Omega-3s are thought to have anti-inflammatory effects and impact neurotransmitter function (49, 50). However, further investigations are needed to fully clarify the potential mechanisms linking specific nutrients to improved mental health outcomes.

As mentioned previously, the specific dietary factors that may influence the association between nutrition knowledge and GAD were not examined. Further studies are recommended to investigate these important factors to better understand how dietary habits, food intake patterns, and overall nutritional quality can mediate the relationship between nutrition knowledge and anxiety. It is also important to acknowledge, understand and investigate the interplay between several factors that were not measured in the present study, but may mediate such associations, such as physical activity (51). For instance, it is possible that individuals with higher nutrition knowledge may engage in more physical activity, which could independently lower anxiety levels (52). Additionally, socioeconomic status, access to healthy foods, and cultural attitudes toward diet could all influence the relationship between nutrition knowledge and anxiety. Thus, the absence of this variable limits our ability to draw definitive conclusions about the direct impact of nutrition knowledge on anxiety disorders. By examining these variables, future research can provide a more holistic understanding of how various lifestyle and contextual factors contribute to mental health outcomes, ultimately leading to more effective interventions.

Given the substantial mental health burden facing societies, and the growing evidence that proper nutrition may offer protective effects, it might be recommended to consider interventions centered on improving nutritional knowledge and healthy dietary habits, as they may serve as an important strategy for supporting population health and mental wellbeing. It is important to mention that several campaigns were launched in the KSA since 2019 as part of Saudi Vision 2030. These initiatives aim to enhance nutrition awareness among the population (53). However, there may be a need for broader dissemination of these initiatives. For instance, it might be recommended to align nutrition education programs with existing health campaigns and initiatives under Saudi Vision 2030. Additionally, it may be beneficial to extend health awareness campaigns to large-scale entertainment events to reach a wider population. Partnering with influencers or local health professionals could further help increase the reach and credibility of nutrition education messages, making them more effective in engaging the target audience.

Using the present-day digital advancement may help disseminate nutrition education initiatives. The use of technology, particularly the internet and handheld smart devices, has become widespread in recent years (54). It might be recommended to also utilize popular social media platforms in Saudi Arabia, such as WhatsApp, Instagram and Snapchat, to share nutrition education content (55). It is also advisable to develop interactive applications that incorporate engaging elements like quizzes, meal planning tools, and gamification techniques. Additionally, these apps can include features that allow users to commit to healthy dietary habits and avoid unhealthy ones. Such functionalities can enhance user engagement, improve nutrition knowledge, and ultimately lead to better dietary choices (56). This technological landscape presents cost-effective opportunities for supporting the dissemination of nutrition education interventions to various groups within the population (57, 58).

Indeed, digital interventions have shown promise in improving nutrition knowledge and promoting healthier lifestyles across different populations. In support of this, a review of 27 studies found that website-based programs, smartphone apps, online courses, and text messages were effective in increasing participants' understanding of nutritional concepts (59). Key factors contributing to the success of these digital interventions included personalized feedback and active engagement between participants and program facilitators. However, the evidence regarding the effectiveness of digital healthy lifestyle interventions within the KSA is limited and inconclusive (60, 61). This paucity of research in the regional context obscures our understanding of the specific factors that may encourage participation in such programs among the Saudi population. Therefore, it is recommended to investigate the factors that may motivate and enable young adults in the KSA to engage with digital nutrition education programs. Also, it is essential to ensure that the development of educational materials accurately reflects traditional Saudi foods and dietary practices. This alignment enhances the relatability and applicability of the content for the target audience. Respecting these contextual and cultural considerations could inform the development and implementation of effective, tailored interventions that holistically address the overall health and wellbeing of the Saudi population.

Finally, the study identified an inverse association between high nutrition knowledge and lower levels of anxiety among a sample of young Saudi adults. These findings may have broader implications for public health policy beyond the framework of Saudi Vision 2030. Specifically, they suggest that enhancing nutrition education may serve as a viable intervention for reducing anxiety levels. Policymakers in various contexts can leverage these insights to develop and implement strategies that incorporate nutrition education into mental health initiatives, potentially improving health outcomes across diverse populations.

## 5 Strength and limitations

The main strength of the present study lies in the fact that it provides the first exploration of the association between nutrition knowledge and GAD among young adults in the KSA, which helps address a significant public health concern within the framework of Saudi Vision 2030. However, the study also has some limitations. The snowball sampling technique employed in this study may introduce potential selection bias. Participants recruited through social media may not adequately represent the general population, which could affect the generalizability of the findings. Additionally, the reliance on self-reported data through online surveys may introduce biases, such as social desirability or recall bias. The study population is also limited to young adults in Saudi Arabia, which may limit the generalizability of the findings to other age groups or geographical contexts. Future research should explore alternative sampling methods and broader demographics to enhance representativeness and reduce these biases.

The sample also demonstrated a gender imbalance, with a higher number of female participants than male. This discrepancy limits the generalizability of the findings to both genders. It is essential for future research to include a more balanced sample to ensure adequate representation of both sexes. Moreover, the cross-sectional design can only establish associations between nutrition knowledge and GAD, not causal relationships. Therefore, future studies are recommended to employ a longitudinal design in a wider range population, and track changes in participants' nutrition knowledge and GAD levels over time, examining the temporal ordering and directionality of their relationship. It is also recommended to establish nutrition educational interventions with a control group to directly test the causal effect of improving nutrition knowledge on the development or reduction of GAD symptoms, as well as determining the efficacy of targeted nutrition education programs in influencing mental health outcomes.

## 6 Conclusion

Considering the goals and objectives outlined in Saudi Vision 2030 to enhance overall health and psychological wellbeing, this study was the first to explore the association between nutrition knowledge and GAD among young adults in Saudi Arabia. The findings indicated that the higher nutrition knowledge level is associated with lower scores of GAD. This highlights the importance of promoting nutrition education and interventions aimed at improving dietary habits as a potential strategy to address the burden of GAD for this population. Further longitudinal and interventional studies are needed to establish the causal relationship between nutrition knowledge and mental health outcomes.

## Data Availability

The raw data supporting the conclusions of this article will be made available by the authors, without undue reservation.
